# Spectrum of Cardiac Arrhythmias in Isolated Ventricular Non-Compaction

**DOI:** 10.19102/icrm.2017.080701

**Published:** 2017-07-15

**Authors:** Alexis Z. Tumolo, Duy T. Nguyen

**Affiliations:** ^1^Division of Cardiac Electrophysiology, University of Colorado School of Medicine, Aurora, CO

**Keywords:** Arrhythmia, atrial arrhythmia, atrial fibrillation, ventricular arrhythmia, ventricular non-compaction

## Abstract

A wide spectrum of cardiac arrhythmias has been observed in patients with isolated ventricular non-compaction, which is defined by hypertrabeculated ventricular myocardium with deep intertrabecular recesses, in the absence of concomitant congenital heart disease. In this genetically diverse phenotype, the development of fibrosis contributes to an arrhythmogenic substrate underlying atrioventricular conduction diseases, supraventricular tachycardias and ventricular tachycardias. Within this spectrum, monomorphic ventricular tachycardia is the most frequently observed arrhythmia, and this prevalence has important implications for sudden cardiac death risk.

## Introduction

Isolated left ventricular non-compaction (LVNC) is a cardiomyopathy defined by hypertrabeculation of the ventricular myocardium, and was first described in 1990.^1^ The clinical course of this cardiomyopathy often includes the development of systolic dysfunction with heart failure symptoms, mural thrombosis and systemic embolism, and cardiac arrhythmias. Specifically, LVNC exists in the absence of other congenital heart defects.^2^ This rare cardiomyopathy has a reported prevalence of 0.05% to 0.25% in adults.^3^ A wide spectrum of cardiac arrhythmias has been described in case reports and series, which are discussed in this article.

### Diagnostic criteria

In the first description of LVNC, published in 1990, Chin et al. examined a series of eight patients with a mean age of 8.9 years, ranging from 11 months to 22.5 years in age, with isolated LVNC **(Table 1)**. The criteria for the diagnosis of LVNC of left ventricular myocardium on transthoracic echocardiogram (TTE) were: (1) the presence of many excessively prominent trabeculations; and (2) an absence of coexisting cardiac abnormalities. The ratio of compacted (epicardium to trough of trabeculations) to non-compacted myocardium (trough to peak of trabeculations) was evaluated at the levels of the mitral valve, papillary muscles, and apex at end-diastole, and compared with normal values for patients in the same age range; LVNC was defined by the ratio of compacted to non-compacted tissue ≤ 0.5.^1^

The definition of LVNC was further refined by Jenni et al. in the late 1990s to include the presence of a thin, compacted epicardal layer and a thicker, non-compacted endocardial layer. Measurements were performed in end-systole from parasternal short-axis and apical four-chamber echocardiographic views, with LVNC defined by a ratio of non-compacted to compacted tissue > 2. Additionally they stipulated that a diagnosis of LVNC requires the absence of other congenital cardiac abnormalities.^2,4,5^

Cardiac magnetic resonance imaging (MRI) provides higher resolution evaluation of the myocardium. Based on a study of patients with established LVNC by Petersen et al., the criteria relied upon by cardiac MRI for diagnosis-include a non-compacted to compacted myocardium ratio of > 2.3 in diastole (sensitivity, 86%; specificity, 75%; positive predictive value, 75%; negative predictive value, 99%).^6^

## Pathophysiology

Non-compacted LV myocardium is a remnant of embryological development. In the first gestational trimester, the heart tube loops to form into a four-chambered heart. The heart tube tissue consists of an inner endocardial cell layer and an outer myoepicardial cell mantle, as well as acellular cardiac jelly. Once the tube has looped to form a primordial ventricular, trabeculations of differentiating cardiomyocytes interspersed with deep intertrabecular recesses form in the cardiac jelly in order to provide more surface area for gas exchange for the developing tissue prior to the formation of epicardial coronary arteries. As the coronary arteries develop between the fifth and eighth week of gestation, the myocardium gradually becomes compacted into the ventricular wall. The process of compaction is generally more complete in LV tissue, leaving the mature right ventricle (RV) more trabeculated. Non-compaction results from a defect in this process.

While the mechanism of systolic dysfunction in LVNC is still not fully understood, it is hypothesized that non-compacted myocardium receives less blood flow from epicardial coronary arteries, leading to adverse remodeling over time.^1,2^ Inadequate blood flow to non-compacted myocardium may contribute to the development of cardiac arrhythmias. Cardiac fibrosis may interfere with the conduction system, leading to conduction delays, and reentrant circuits may develop from the border zones of fibrosis and normal myocardium. In a series of five children, aged 10 to 14 years old, Junga et al. identified evidence of non-compaction, and studied blood flow at rest with position emission tomography (PET) with [^13^N]-ammonia, and at stress with intravenous dipyridamole. In this group, PET images taken at rest demonstrated a 16% to 33% impairment, while PET images taken during stress demonstrated a 32% to 57% impairment, in blood flow to non-compacted myocardium, in comparison with compacted myocardium. While their patients experienced no significant arrhythmias on Holter monitoring, late potentials were seen in the resting electrocardiograms (ECGs) of three patients, and prolonged QT dispersion was identified in one patient. The authors posited that arrhythmias observed in other studies may be secondary to inadequate micro-circulation to the non-compacted myocardium.^7^

### Genetic basis

Multiple genetic mutations have been implicated in the LVNC phenotype. Oechslin and Jenni describe mutations in mitochondrial, cytoskeletal Z-line and sarcomeric proteins, which are linked to autosomal-dominant, autosomal-recessive, and X-linked inheritance. In the pediatric population, mutations in the *G4.5* gene on chromosome Xq28 are commonly seen in LVNC patients; mutations in the same gene are often associated with the onset of other cardiomyopathies, such as dilated cardiomyopathy in Barth syndrome. However, *G4.5* mutations are not observed in the adult population, who demonstrate autosomal-dominant mutations more often. Patients with LVNC and LV systolic dysfunction may have mutations in alpha-dystrobrevin and in *ZASP,* a gene encoding Z-band protein; however, these are not observed in cases of isolated LVNC without systolic dysfunction. Furthermore, genetic mutations associated with LVNC have also been mapped to chromosomes 1, 5, and 11. Mutations in genes encoding sarcomeric proteins, which have been observed in other cardiomyopathies, are also described in cases of LVNC; these include a missense mutation in pE96K in the troponin T gene.^8^ LVNC has also been observed in conjunction with inherited long QT syndrome secondary to *KCNH2* mutations; Ogawa et al. described one pediatric patient with an A561V mutation in the pore region, and another with a D501N mutation in the non-pore region.^9^ Furthermore, it has been associated with the ryanodine receptor gene *(RYR2)* exon 3 deletion that is described to be a causative mutation in catecholaminergic ventricular tachycardia (CPVT) and in dilated cardiomyopathy. In a series published by Ohno et al., two unrelated individual CPVT probands, each with large RYR2 exon 3 deletions, and concomitant LVNC, were identified. Among the relatives of these probands, eight of the carriers of the deletion were also diagnosed with LVNC.^10^

### ECG abnormalities

A variety of baseline ECG abnormalities have been reported in patients with LVNC. These are summarized in **Table 2**, and are also reviewed later in this article in the context of various observational studies. ECG abnormalities, however, are not the focus of this review.

## The overall spectrum of arrhythmias

In Chin et al.’s pivotal description from 1990, the series of eight patients suggested that arrhythmias could occur on a wide spectrum in LVNC patients **(Table 3)**. Five of the eight patients had ventricular arrhythmias, which manifested as isolated premature ventricular contractions (PVCs) in two patients; ventricular tachycardia (VT) in two patients; and supraventricular tachycardia (SVT) with atrioventricular (AV) reentrant tachycardia that led to ventricular fibrillation (VF) in one patient. Additionally, one patient demonstrated complete heart block requiring resuscitation and permanent pacemaker placement, and two patients had resting heart rates of less than 40 bpm. On ECG, axis deviation was present in two patients, P-wave abnormality was present in three, first-degree AV block was present in two, and LV conduction defects were present in two.^1^

A large series of LVNC patients was followed by Oechslin et al. Specifically, they described the course of 34 adults, with mean age at diagnosis 42 ± 17 years, over 44 ± 39 months. In this patient group, the following electrophysiologic abnormalities were observed: abnormal ECG (n = 32, 94%); VT (n = 14, 41%); chronic atrial fibrillation (AF) (n = 9, 26%); right bundle branch block (RBBB) (n = 4, 12%), left bundle branch block (LBBB) (n = 15, 44%); and repolarization abnormality without block (n = 12, 35%). Wolff–Parkinson–White (WPW) syndrome, though looked for, was not observed in any patients (n = 0, 0%). During this prospective series, 12 patients (35%) died, and four underwent cardiac transplantation.^5^

Lofiego et al. performed a retrospective analysis of 65 adult patients noted to have isolated ventricular non-compaction by TTE; these patients were followed up with for six to 193 months (a mean of 46 months). For 48 (74%) of these patients, diagnosis was based on symptoms, while in the cases of 17 (26%) patients, diagnosis was not symptom-based, but rather based on family referral, ECG abnormalities, elevated biomarkers, and the presence of heart murmur. In the group of patients with a symptom-based diagnosis, sustained VT occurred in four (8%), syncope in three (6%), permanent AF in six (13%), and sudden death in three (6%) patients over the course of follow-up. Interestingly, none of these arrhythmias were found in patients in the group without symptoms. Additionally, over the course of follow-up, the symptom-based group had 15 (31%) instances of cardiovascular-related deaths or heart transplantation, as compared with zero (0%) in the group without symptoms. In univariate and multivariate analysis, sustained VT was a significant predictor of death or transplantation, while permanent AF and syncope were not significantly associated with these outcomes.^11^

In a previous systematic review, Bhatia et al. compiled data from five cohorts of patients with LVNC to create a sample size of 241 patients. They cited data from five studies, in which Holter monitoring was performed symptom-driven in one study and routinely in four. From the data pooled from these studies, AF was found in 23 patients (n = 23/241, 10%); non-sustained VT (NSVT) in 36 patients (n = 36/106, 33%), sustained VT in eight patients (n = 8/171, 5%); and unspecified VT in four patients (n = 4). The authors further reported that 26 patients (11%) had an implantable cardioverter-defibrillator (ICD) placed for VT (n = 10, 4%); presyncope with inducible VT on electrophysiology (EP) study (n = 2, 0.8%); and primary prevention of sudden cardiac death (n = 14, 6%). Additionally, they noted that one patient died of refractory VT, that two survived appropriate ICD discharges, and that two were inappropriately shocked for atrial dysrhythmias.^12^

While this article has primarily focused on reviewing arrhythmias in adult patients in LVNC, it is important to note that cardiac arrhythmias can manifest in ILNC patients during childhood, with slightly different phenotypes. In a series of pediatric patients with LVNC, the incidence of arrhythmia was high, though the proportion of ventricular arrhythmias was lower. Ichida et al. conducted a nationwide survey of 150 Japanese hospitals, identifying 27 patients with confirmed LVNC by echocardiography for inclusion in the study, with ages ranging from one week to 15 years at presentation. Follow-up for these patients extended for as long as 17 years after presentation. Twenty-two (88%) of these patients had baseline abnormalities on ECG, including ST depression, T-wave flattening or negative T-waves in the inferior and lateral precordial leads, and RBBB, in addition to QRS axis deviation and evidence of chamber enlargement. The authors noted that there were fewer instances of LBBB than was seen in adults with LVNC. Three patients had WPW syndrome, and a fourth had a concealed accessory pathway involved in SVT. Over the course of follow-up, 13 (48%) patients had significant arrhythmias, including paroxysmal SVT (n = 2, 7%); AF (n = 1, 4%); PVCs (n = 7, 26%); second-degree AV block (n = 2, 7%); and complete heart block (n = 1, 4%). This cohort demonstrated no sustained VT or NSVT.^13^

### Bradyarrhythmias

Bradyarrhythmias from sinus node dysfunction to progressive degrees of AV delay and block have been noted in LVNC patients. Chin et al. reported two patients with significant sinus bradycardia, two patients with first-degree AV block, and one patient with complete heart block in their series.^1^ The cohort followed by Ichida et al. also included patients with bradyarrhythmias. Of the 27 patients with LVNC included in the study, two developed second-degree AV block, and one had complete heart block.^10^ In addition to these, two case reports have described bradyarrhythmias.

A case of a patient presenting with complete AV block as a first manifestation of LVNC non-compaction was described by Nascimento et al. This patient had dyspnea on exertion, as well as prescyncope, which progressed to four episodes of syncope with Holter monitoring, revealing sinus bradycardia with AV delay, second-degree Mobitz II AV block, and episodes of complete AV block, requiring placement of a permanent pacemaker. Her TTE confirmed LVNC by the presence of numerous and prominent trabeculations.^14^

Jackson et al. similarly described the case of a patient diagnosed with non-compaction after the occurrence of complete atrioventricular block with syncope. After non-compaction was diagnosed on TTE, this patient had a dual-chamber ICD implanted, and went on to develop ventricular tachycardia.^15^

### Supraventricular tachycardia

SVT tends to be more prevalent in children with LVNC, though AF is reasonably common in adult patients, as reported in the Chin et al., Oechslin et al., and Lofiego et al. series.^1,5,8^ Notably, SVTs described in this patient population include AF and atrial tachycardia (AT).

In a case report by Kato et al., a 19-year-old man with a first presentation of AT that occurred during childhood suppressed with β-blockers re-presented with presyncope associated with episodes of rapid AF followed by VT, which was demonstrated on Holter monitoring. Echocardiogram demonstrated prominent trabeculations and intertrabecular recesses in the LV, together with mild LV dilation and mildly reduced LV fractional shortening, consistent with non-compaction. During EP study, AF and AT were easily triggered by isoproterenol infusion, and VT followed these rhythms, requiring multiple shocks. The pulmonary veins were isolated, and subsequently neither AF nor VT was inducible. The authors did not comment on whether this patient received an ICD. He had no further SVT episodes during a follow-up period of three years.^16^

Alihangolu et al. described a 25-year-old woman with a history of palpitations, sweating and shortness of breath for two months who was found to have non-compaction with an LV ejection fraction (EF) of 32%. The patient’s presenting rhythm was AF with rapid ventricular response.^17^

### Ventricular arrhythmias

Ventricular arrhythmias have been described more frequently in adults with LVNC. Monomorphic VT, polymorphic VT, bidirectional VT, and VF have all been cited several times, and together raise concerns regarding the higher risk of sudden cardiac death in this patient population.

***Monomorphic VT.*** In Oechslin’s series of 34 patients with LVNC, ventricular tachycardias were demonstrated in 14 patients (41%), with NSVT seen in 11 and sustained VT seen in three. Though one patient died within an hour of VT onset, ICDs were successfully implanted in four patients, specifically for sustained VT in two, and for presyncope with inducible VT by EP study in two. One patient with an ICD died from refractory VT.^5^

Fazio et al. followed a series of 238 consecutive patients with LVNC from the Italian Society of Echocardiography registry between May 2005 and September 2006, using serial monitoring. Patients were followed for a mean period of 4.13 years (range, one to 12 years), during which time, clinical evaluation, ECGs and 24-hour Holter monitoring were performed every six months. The investigators observed that 11 patients (4.3%) in this cohort had VT on Holter, with sustained VT noted in two patients. Patients with VT were noted to have a mean LV EF of 43.72%, with a range of 16% to 60%. One patient was a three-year-old male child with fascicular VT, which was ablated successfully. Over the course of the follow-up, an ICD was implanted in one patient, and there were no deaths. Patients with observed VT were treated with β-blockers.^18^

In a case series of 25 patients with a personal history of VT, along with a family history of sudden cardiac death, in the absence of angiographically significant coronary artery disease, cardiac MRI was performed for further evaluation. In this series, MRI revealed non-compaction in three patients.^19^ In a separate case report, Alihanoglu et al. described a 25-year-old woman with AF at the time of LVNC diagnosis who had sustained VT induced during her EP study, and who required amiodarone for cardioversion and hemodynamic stabilization. Given her reduced LV EF, she was optimized on goal-directed medical therapy; an ICD was implanted prior to discharge.^17^

Non-sustained monomorphic VT arising from the LV has been described. Vijayvergiya et al. detailed the case of a 32-year-old man who was found to have asymptomatic salvos of NSVT with RBBB morphology. TTE at the time of diagnosis showed non-compaction with an LV EF of 30%. The patient’s tachycardia was successfully suppressed with amiodarone, but ICD implantation was deferred due to financial constraints.^20^

**Figure 2:**
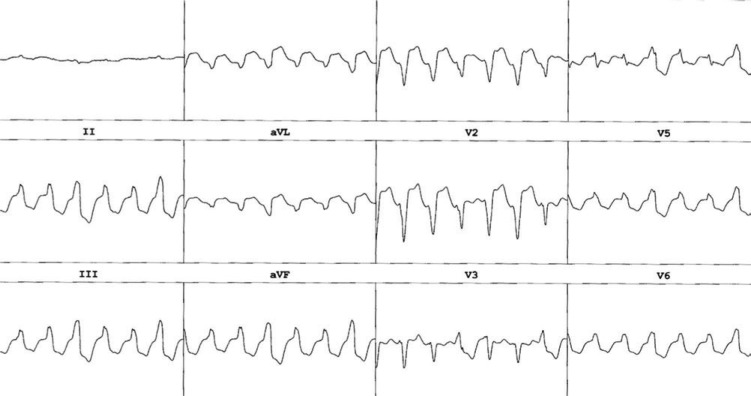
A 12-lead ECG of monomorphic VT, seen in a patient with biventricular non-compaction.

**Figure 3:**
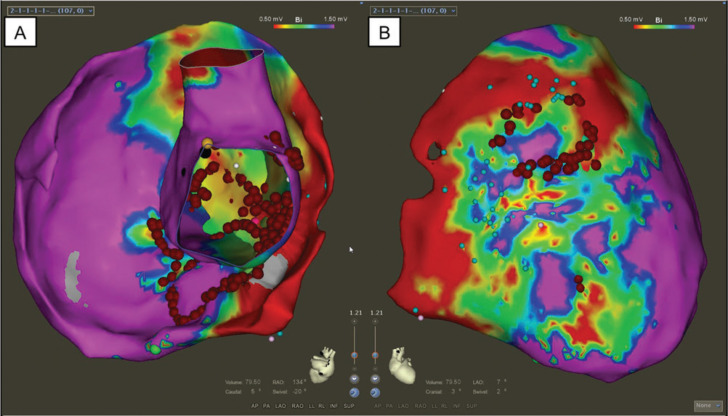
**A:** A 3D CARTO (Biosense Webster, Diamond Bar, CA) electroanatomic map with an internal view of the RV epicardial shell with RV endocardial shell superimposed on it with ablation lesion tags (red circles) in a patient with biventricular non-compaction. Bipolar voltage scale is from 0.5 to 1.5 mV with < 0.5 mV as dense scar and > 1.5 mV as normal voltage. **B:** The epicardial surface with ablation lesion tags.^20^

VT in a structurally normal RV has been reported in association with a non-compacted LV. Honarbaksh et al. described the case report of a 67-year-old man with established isolated LVNC. He also had a history of RV outflow tract (RVOT) ventricular extrasystoles status post-ablation in the past, but had developed new ventricular ectopy and runs of NSVT from the basal RV localized to the inferolateral tricuspid annulus. By cardiac MRI, the RV free wall displayed no evidence of non-compaction. It is possible that the occurrence of monomorphic VT from the RV is unrelated to this patient’s underlying non-compaction. However, the authors stipulate that the two processes may have been related—namely, that MRI may not have been sensitive enough to detect RV trabeculations, or that the VT may have resulted from an accessory pathway that regressed.^21^ This case is similar to another case reported by Gonzalez et al., where a 19-year-old patient had LVNC diagnosed by cardiac MRI and who developed an epicardial VT from the RV. A 12-lead ECG of her VT is displayed in **Figure 2**, and voltage mapping performed to guide her ablation is shown in **Figure 3**.^22^ In both of these cases, as well as in the next, it is noted that RV non-compaction (RVNC) is less frequently described; normal RV myocardium can be highly trabeculated, which may decrease identification. Additionally, Osmonov et al. also described a patient with VT originating from the RVOT in conjunction with LVNC. This patient was a 16-year-old male who was noted to have frequent PVCs with left bundle branch (LBB) morphology, inferior axis, and precordial transition at V4. His TTE showed an LV EF of 29%, with non-compaction of the apical and lateral LV walls. During EP study, his VT was pace-mapped to the anteroseptal RVOT, and was successfully ablated using radiofrequency (RF) energy. Two months after the ablation, non-compaction persisted, but his LV EF had improved to 53%; there were no PVCs observed on 24-hour Holter monitoring.^23^

In a short case report, Santoro et al. described a patient with LV non-compaction afflicted by monomorphic VT of multiple morphologies. This patient was a 74-year-old man who presented with palpitations, and who was found to have monomorphic VT with LBB morphology and right inferior axis. His TTE showed his LV EF was depressed to 40%, with non-compaction of both the LV and RV. No EP study was performed, but the patient was discharged with an ICD and on amiodarone.^24^

Ablation of VT in LVNC has been described in several patients as a treatment for VT and PVCs. Lim et al. presented a patient who underwent successful endocardial and epicardial mapping, as well as RF ablation, for monomorphic left VT. A 52-year-old man with a prior history of presyncope and palpitations was admitted with palpitations and dizziness, and was found to have VT with a RBBB, right inferior axis. Echocardiogram demonstrated LVNC with an LV EF of 30%. In the EP laboratory, VT was easily inducible, and was mapped to the anterolateral LV, with possible epicardial origin. Therefore, simultaneous endocardial and epicardial mapping was done, with radiofrequency ablation performed at the site of earliest activation on the anterolateral LV epicardium. Ablation was successful, and the patient remained free of VT during the following eight months.^25^

Jackson et al.^15^ described a case of refractory VT in a patient with non-compaction that went on to receive catheter ablation. Their patient was an 18-year-old male who was diagnosed four years prior with mild LVNC involving the free wall and distal septum and associated with an LV EF of 45% to 55%; he had undergone dual-chamber ICD implantation at the time of diagnosis, due to syncope with noted complete heart block, described previously. He went on to experience symptomatic sustained and non-sustained monomorphic and polymorphic VT requiring multiple shocks and antiarrhythmics over the following four years. Owing to refractory VT, he underwent substrate-based electroanatomic mapping of three distinct VTs in the left ventricle, and ablation of the best pace-matches as well as the sites with fractionated and late potentials with 46 RF lesions. Over the following 12 months, the patient experienced only a single four-beat run of NSVT.^12^

Paperella et al. allude to nuances in the evaluation of sudden cardiac death (SCD) risk for patients with non-compaction in a case report of a 59-year-old man admitted with palpitations who was found to have a wide-complex tachycardia with right bundle branch morphology, left superior axis, consistent with a VT from the LV, which was suspected to be originating from the posterior papillary muscle. After TTE showed a dilated LV with reduced EF < 25% and hypertrabeculated endocardium in the LV apex, with non-compacted to compacted myocardial ratio consistent with non-compaction, the patient underwent an EP study, during which no sustained SVT or VT was inducible; furthermore, pace mapping of the LV demonstrated no match to the clinical VT, and voltage mapping of the LV revealed no abnormalities. No ablation was performed, but an ICD was implanted at that time because of the presence of a past medical history of syncope in addition to the VT documented on presentation.^26^

Recently, Muser et al. identified nine patients with known LVNC amongst a cohort of 1,816 patients with VT or frequent PVCs who underwent RF ablation. Seven of the nine patients in the series had frequent PVCs, and two had VT storm. It was noted that focal PVCs tended to arise from the LV basal-septal regions and papillary muscles, while VT involved mid-apical LV segments, matching the non-compacted myocardial segments. Catheter ablation was successful in eight patients. The VT patients had no recurrence at four and seven months of follow-up, and those patients with PVCs displayed a significant reduction in PVC burden.^27^

***Bidirectional VT.*** Two patients have been described as having bidirectional VT in the LVNC literature. In a short imaging report, Arias et al. detailed the case of a 29-year-old woman with LVNC evidence from cardiac MRI who developed bidirectional VT. She initially presented with a wide-complex tachycardia consistent with a left bundle, right inferior axis with transition at V5; this was terminated with cardioversion. During invasive EP study, a single extra stimulus at the RV apex initiated bidirectional VT, with one morphology identical to the presenting rhythm, and the other a left bundle, right superior axis with transition at V6.^28^

Fiala et al. described a case of bidirectional VT from a deep septal focus. In this case, a 17-year-old male who had been diagnosed with biventricular non-compaction four years prior with reduced LV EF and symptoms of heart failure experienced a bout of VT that was unable to be controlled with antiarrhythmic medications. He underwent electroanatomic mapping and ablation of his clinical LBBB morphology VT over the RV septum adjacent to the insertion of trabecular septomarginalis, but he subsequently developed a second RBBB morphology VT. Repeat mapping and ablation were performed from the LV over the septum, but this was limited by steam pops. Repeat ablation was performed by the RV septal side with successful suppression of all VT on β-blocker therapy alone.^29^

***Polymorphic VT.*** Cases of polymorphic VT in LVNC have been reported, possibly in conjunction with underlying genetic channelopathies.

In a short communication, Seres et al. presented a case of polymorphic VT in a 57-year-old woman with a family history of SCD, with a presenting symptom of syncope. Upon evaluation, she was found to have a LV EF of 46%, with evidence of non-compaction by prominent trabeculations and deep intertrabecular recesses in her anterior and lateral apical wall segments on TTE, and a prominent trabecular zone and deep intertrabecular recesses in the anterior wall by left ventriculography. Telemetry showed multiple episodes of polymorphic VT. Invasive EP study revealed a prolonged HV interval at 62 ms; programmed ventricular stimulation with up to three extrastimuli from the RV apex failed to induce ventricular arrhythmias. Given that polymorphic VT persisted despite the use of β-blockers, and that the patient at baseline had prolonged HV conduction, a dual-chamber ICD was implanted. There was no evidence of an inherited long QT syndrome being present on ECG screening of the patient’s living immediate relatives.^30^

Polymorphic VT in the context of prolonged QT due to *KCNH2* mutation was described in two case reports by Ogawa et al. The first discusses a one-day-old male who, 13 hours after birth, developed tachypnea, tachycardia, hypotension, and cyanosis accompanied by metabolic acidosis and hypocalcemia; his ECG showed LBBB with prolonged QT. Following calcium repletion, he developed polymorphic VT, which was treated with lidocaine, cardioversion, and magnesium. Subsequent ECGs and Holter monitoring showed both narrow QRS morphologies and LBBB intermittently. An echocardiogram demonstrated hypertrophic trabeculations, mostly in the apical LV endocardium. His genetic analysis was significant for *KCNH2* (LQT2) missense mutation A561V. The second case involved a five-year-old male with a seizure disorder who was found to have prolonged QT at 590 msec. Holter monitoring demonstrated T-wave alternans and Torsades de pointes. An echocardiogram revealed prominent trabeculations in the apical, lateral and inferior LV, with preserved EF of 74%. The patient’s genetic analysis was also significant for a mutation in *KCNH2* with D501N. The authors raise the concern regarding the higher risk of SCD in such patients, and recommend the implantation of an ICD.^31^

Campbell et al. described an interesting case of CPVT occurring in a patient who developed LVNC, and who was found to have deletion of exon 3 of the *RYR2* gene encoding the ryanodine receptor. This patient suffered multiple expertional syncopal episodes as a child and adolescent, with one episode occurring with documented AF. When her AF was recognized after syncope at age 16, a TTE showed new trabeculations in the LV apical and posterior free walls. She subsequently experienced an episode of VF cardiac arrest during exertion one year later, and was found to have LVNC. After that event, an ICD was implanted. Four more instances of VF cardiac arrests occurred, and the patient was treated with multiple antiarrhythmic agents, followed by a bilateral sympathectomy. On comprehensive genetic testing, this patient was found to have deletion of the *RYR2* gene, which has been reported in other cases of CPVT. This patient’s course is significant, as her time course of worsening arrhythmias coincided with the development of LVNC, which was not present on TTEs at the times of her initial syncopal episodes. Furthermore, it raises the question of whether her CPVT and LVNC may both be associated with her genetic mutation.^32^

***VF.*** VF in the context of LVNC has been reported in three children and one adult. Although ventricular arrhythmias are rarer in pediatric patients, Celiker et al. described the clinical course of a six-year-old female with recurrent syncope whose baseline ECG showed biphasic T-wave inferiorly and laterally. She was found to have evidence of non-compaction on TTE, with a preserved EF of 65%. After presentation with VF, she had recurrence of VF that required amiodarone and several defibrillations. She underwent ICD implantation, and was discharged on amiodarone and propranolol. Three episodes of VF were treated by her ICD in the following 16 months.^33^

Additionally, Ergul et al. documented the case report of a four-year-old male with recurrent syncope in the prior year who was admitted after aborted SCD; his ECG showed non-specific ST-T changes, and TTE revealed non-compaction. During EP study, polymorphic VT was induced by programmed ventricular stimulation, and an epicardial ICD was implanted. Five months after discharge, he experienced VF, for which he received an appropriate shock.^34^

Cohen and Prahlow describe SCD in a 14-year-old African-American girl who was found to have LVNC. She had a history of “episodic tachycardia,” which had been treated with ablation eight years prior. With cardiac arrest, she had seizure-like activity, and was noted to be asystolic on arrival, with multiple subsequent defibrillations for VT and VF needed during resuscitation over the course of 5.5 hours. Autopsy revealed biventricular dilatation with LV apical non-compaction and subtle RVNC; it does not appear that the diagnosis of non-compaction was known prior to autopsy.^35^

Sato et al. described an instance of cardiac arrest because of VF in a 57-year-old previously healthy man who was found to have flattened T-waves in the lateral precordial leads on ECG and an LV EF of 58%; cardiac MRI showed non-compacted myocardium in the lateral and apical walls. Subsequent EP study was notable for the presence of late diastolic potential, and programmed ventricular stimulation from the RV apex with triple extrastimuli induced VF. After implantation on an ICD, the patient had one episode of VF that was treated in the subsequent eight weeks.^36^

## Assessing SCD risk with EP study

Based on these reports of ventricular arrhythmias, it can be inferred that patients with LVNC are at a higher risk for SCD. Ritter et al. reported a 47% incidence of ventricular arrhythmias in non-compaction, with these arrhythmias accounting for half of the deaths in this patient population; furthermore, it seems that fatal ventricular arrhythmias occur more frequently in those with decreased LV EF.^37^ Several case reports and series have detailed experiences in using invasive EP studies to risk stratify patients.

In order to identify patients at risk for ventricular arrhythmias, Steffel et al. studied the prognostic significance of findings during EP study. A retrospective analysis of EP study findings and clinical follow-up for 24 patients with LVNC at two Swiss centers was performed. During EP study, with ventricular extrastimuli and isoproterenol infusion, ventricular arrhythmias were inducible in nine patients (38%); two had sustained monomorphic VT, two had sustained polymorphic VT or VF, and two had non-sustained polymorphic VT. Of the nine patients, seven underwent ICD implantation, and three of these patients went on to experience ventricular arrhythmias over a follow-up period of 61 months; all of these instances of tachyarrhythmia were treated with ATP or shock. Additionally, supraventricular arrhythmias were induced in seven patients (29%), including AF, AV reentrant tachycardia, AV nodal reentrant tachycardia, and atrial flutter. Three patients were noted to have abnormal AV conduction with two having complete heart block (8%) and one having prolonged AV delay.^38^

Additionally, investigators have also followed the outcomes of LVNC patients following ICD implantation to assess the need for therapy: in a retrospective analysis of 12 patients with LVNC who had undergone ICD implantation, Kobza et al. evaluated the rationale for implantation and observed the incidence of appropriate ICD shocks. In two Swiss hospitals from September 1997 to November 2009, 105 patients were diagnosed with LVNC. Based on a patient history of symptomatic arrhythmias or severely reduced LV EF on admission, either secondary or primary prevention ICDs were implanted in 12 patients (eight secondary prevention, four primary prevention). EP study to evaluate for inducible ventricular tachyar-rhythmias was performed in seven patients; ventricular tachyarrhythmias were induced in six patients (86%), which were sustained in three (43%) and non-sustained in three (43%). Single-chamber ICDs were implanted in six patients (50%), dual-chamber ICDs were implanted in four (33%), and biventricular ICDs were implanted in two (16%). During a follow-up period of 1.1 to 98.4 months (median of 36 months), five patients (42%) experienced appropriate ICD therapy (one with ATP, four with ICD shocks); all patients with sustained VT on EP study experienced appropriate ICD therapy. Four of the eight patients (50%) implanted with ICDs for secondary prevention and one of the four patients (25%) implanted with an ICD for primary prevention experienced appropriate ICD therapies. A high proportion of patients with ICDs (eight of 12, 68%) experienced supraventricular tachyar-rhythmias during follow-up. Three inappropriate shocks occurred for sinus tachycardia, AF, and T-wave over-sensing, respectively.^39^

This series of patients was expanded to 30 patients, with the inclusion of cardiac resynchronization therapy (CRT) in a subsequent report from Kobza et al. In this larger group, primary prevention ICDs were implanted in 18 patients, and secondary prevention ICDs were implanted in 12. During the follow-up period of 40 ± 34 months, 11 patients (six primary prevention, five primary prevention; 37%) presented with appropriate ICD therapies. Appropriate therapies occurred after a mean follow-up period of 21 ± 16 months. Furthermore, functional New York Heart Association class improved in the six patients who received CRT as well. The authors concluded that implantation of ICDs in LVNC patients for primary or secondary prevention was appropriate, although the clinical indicators for appropriate ICD therapy were unable to be established.^40^

## Summary

Although LVNC is a rare cardiomyopathy, arrhythmic manifestations of the disease are often significant, and can lead to mortality. Cardiac arrhythmias most likely result from the development of fibrosis in non-compacted myocardium, with the occurrence of the development of conduction delays and reentrant circuits at the border zones of fibrosis and normal myocardium. Arrhythmic manifestations can range from conduction disease to SVT and ventricular arrhythmias. Due to the development of ventricular arrhythmias, these patients are at higher risk for SCD. EP study or the implantation of an ICD should be considered for these patients, especially if they demonstrate signs of any ventricular arrhythmias or a reduced LV EF.

## Figures and Tables

**Figure 1:**
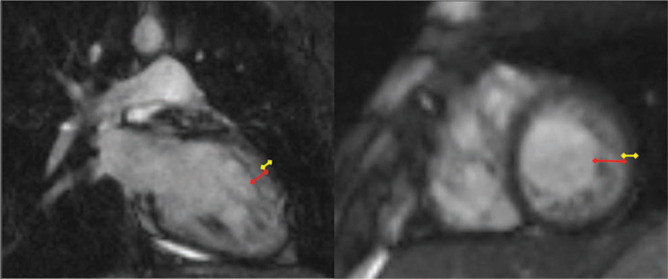
Cardiac MRI images of a patient with IVNC, demonstrating NC:C ratio of 3.7:1 (diagnostic 2.3:1).

**Table 1:** Diagnostic Criteria for Isolated Ventricular Non-Compaction

TTE	
Chin 1990^1^	1. Excessively prominent trabeculations.
	2. NC:C ratio ≥ 2.
	3. Absence of coexisting cardiac abnormalities.
Jenni 2001^4^	1. Thin, compacted epicardial layer with thicker, non-compacted endocardial layer.
	2. NC:C ratio ≥ 2; measured in end-systole from parasternal long-axis and apical four-chamber views.
**MRI**	
Petersen 2005^6^	NC:C ratio > 2.3 in diastole.

TTE: transthoracic echocardiogram; MRI: magnetic resonance imaging; NC: non-compacted myocardium; C: compacted myocardium; MV: mitral valve.

**Table 2:** ECG Abnormalities Reported in Non-Compaction

• Overall abnormal ECG
• AV conduction delay
• P-wave abnormality
• QRS axis deviation
• Evidence of chamber enlargement
• Interventricular conduction defect
• Left bundle branch block
• Right bundle branch block
• ST depression
• T-wave flattening or inversions

**Table 3:** Arrhythmias Reported in Isolated Ventricular Non-Compaction

Arrhythmias	Number of Reported Cases in the Literature Out of a Total of 557 Reported Cases of LVNC*
**Bradyarrhythmias**	
Sinus bradycardia	3
First-degree AV block	3
Second-degree, Mobitz II AV block	3
Third-degree AV block (complete heart block)	6
**Supraventricular tachycardias**	
Atrial fibrillation	22
Atrial flutter	2
Atrial tachycardia	2
Atrioventricular nodal reentrant tachycardia	1
Atrioventricular reentrant tachycardia	6
**Ventricular arrhythmias**	
Premature ventricular contractions	17
Monomorphic ventricular tachycardia	66
Bidirectional ventricular tachycardia	2
Polymorphic ventricular tachycardia	8
Ventricular fibrillation	5

*Excluding the systematic review by Bhatia et al.^12^
